# Supplementary material for: Scalable Solution-Processed Fabrication Approach for High-Performance Silver Nanowire/MXene Hybrid Transparent Conductive Films
Source: Nanomaterials (Basel). 2021 May 21;11(6):1360. doi: 10.3390/nano11061360 (PMC8224074; doi:10.3390/nano11061360)
Supplement: Supplementary file 1 [file nanomaterials-11-01360-s001.zip › nanomaterials-1214071-supplementary.pdf]

nanomaterials

MDPI

Support Information

Scalable Solution-Processed Fabrication Approach for High-Performance Silver Nanowire/MXene Hybrid Transparent Conductive Films

Pengchang Wang, Chi Zhang, Majiaqi Wu, Jianhua Zhang, Xiao Ling and Lianqiao Yang *

Key Laboratory of Advanced Display and System Applications, Ministry of Education, Shanghai University, Yanchang Road 149, Shanghai 200072, China; wangpc@shu.edu.cn (P.W.); zhangchi303145@163.com (C.Z.); wumjq@shu.edu.cn(M.W.); jhzhang@staff.shu.edu.cn (J.Z.); ling_xiao@shu.edu.cn (X.L.)

* Correspondence: yanglianqiao@i.shu.edu.cn; Tel.: +86-21-56333362

Table S1. Typical transmittance and sheet resistance values of AgNWs films before and after coating MXene with different contents.

**Samples**	**Transmittance (%)**			**Sheet Resistance (Ohm/sq)**	**FoM**
	**400nm**	**550nm**	**800nm**		
AgNW	85.8	91.6	89.2	41.4	101.5
AgNW/MXene1	89.9	91.5	89.1	28.2	147.2
AgNW/MXene2-1:20	89.5	90.9	88.6	20.7	177.8
AgNW/MXene2-1:15	86.5	90.5	88.4	20.1	183.2
AgNW/MXene2-1:10	86.5	90.1	87.7	16.9	208.5
AgNW/MXene2-1:7	85.2	89.3	86.7	15.1	214.4
AgNW/MXene2-1:5	82.2	88.1	85.7	14.4	200.2
AgNW/MXene2-1:3	83.5	85.4	81.7	13.6	182.2
AgNW/MXene2-1:1	78.9	82.2	80.0	16.7	109.6
AgNW-MXene(1:7)	89.1	89.8	87.5	19.5	174.9

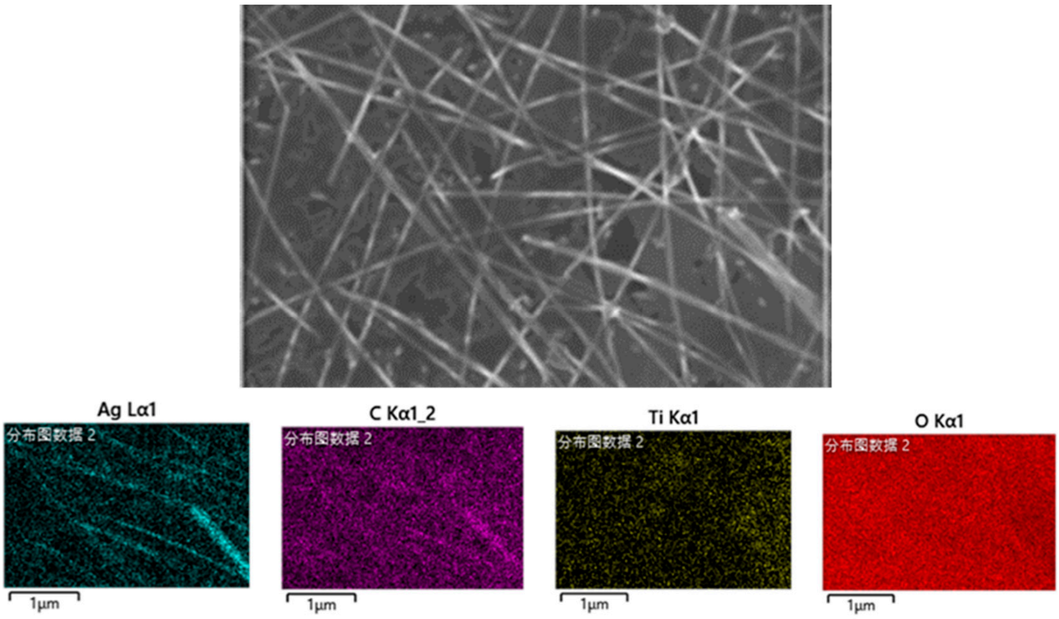

Figure S1. The SEM image and the element analysis of AgNW/MXene2 film.

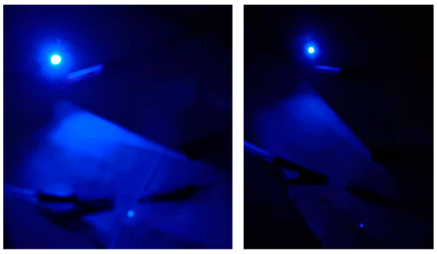

Figure S2. Luminescence experiment of LEDs connected by AgNW/MXene2 and AgNW transparent electrodes with 100mA.

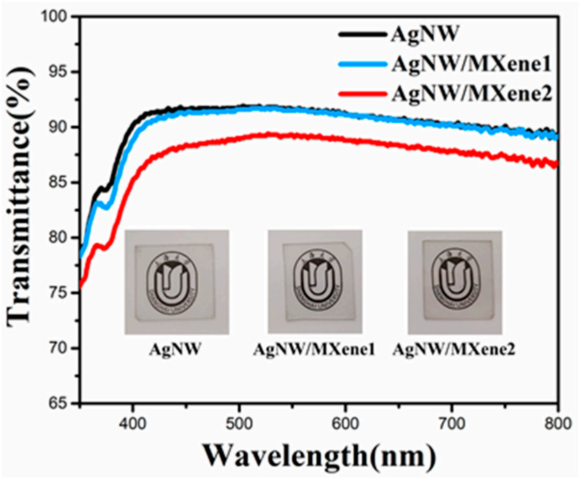

Figure S3. Transmittance spectra of AgNW, AgNW/MXene1, and AgNW/MXene2 TCFs and the photograph of the different films on glass.

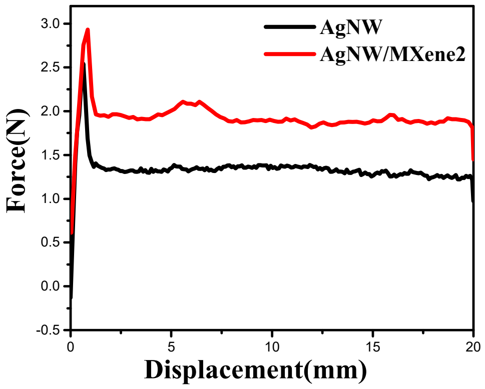

Figure S4. Force–displacement curves for TCFs with and without MXene on glass substrates.

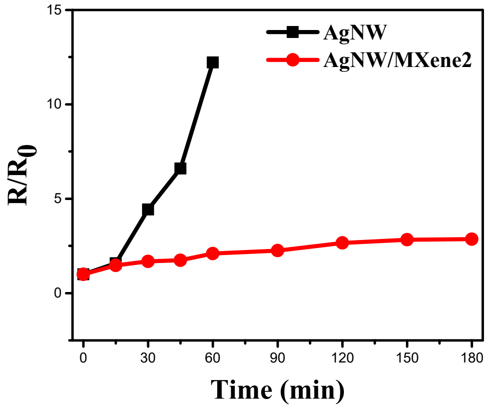

Figure S5. Sheet resistance as a function of time at 180 °C on a hotplate for 180 min.
